# Fusion of Deep Learning Radiomics and Clinical Features for Individualized Prediction of Neoadjuvant Chemotherapy Response in Osteosarcoma

**DOI:** 10.3390/diagnostics16203411

**Published:** 2026-10-20

**Authors:** Jianhua Mu, Yi Luo, Han Liu, Xuanhong He, Minxun Lu, Fan Tang, Yitian Wang, Yong Zhou, Li Min, Chongqi Tu

**Affiliations:** 1 Department of Orthopedics, Orthopedic Research Institute, West China Hospital, West China Medical School, Sichuan University, Chengdu 610041, China; 2 Model Worker and Craftsman Talent Innovation Workshop of Sichuan Province, West China Hospital, Sichuan University, Chengdu 610041, China

**Keywords:** osteosarcoma, deep learning, neoadjuvant chemotherapy, interpretability

## Abstract

**Background:** Stage IIB conventional osteosarcoma exhibits highly heterogeneous responses to neoadjuvant chemotherapy, necessitating reliable, noninvasive pre-treatment evaluation systems. **Methods:** This retrospective study of 198 patients utilized pre-treatment T2-weighted magnetic resonance imaging (MRI) and baseline clinical data. Twelve classical convolutional neural network architectures were benchmarked to identify the optimal imaging backbone. This selected radiomics score was then integrated with core clinical variables using logistic regression to construct a multimodal model. Gradient-weighted Class Activation Mapping++ (Grad-CAM++) and SHapley Additive exPlanations (SHAP) algorithms provided image- and feature-level interpretability. **Results:** DenseNet121 achieved the highest mean cross-validated area under the curve (AUC; 0.775) and was selected, with AUCs of 0.797 (95% CI: 0.716–0.877) and 0.701 (95% CI: 0.513–0.877) in the training and test cohorts. Adding the clinical variables improved discrimination, yielding a test-cohort AUC of 0.827 (95% CI: 0.661–0.992) with significant reclassification gain over the radiomics-only model (NRI = 1.28, IDI = 0.177; DeLong *p* = 0.011). Grad-CAM++ activation concentrated on the solid medullary and extraosseous soft-tissue components, and SHAP ranked the radiomics score as the strongest contributor. **Conclusions:** Integrating deep learning radiomics with key clinical features provides a noninvasive tool for the individualized prediction of neoadjuvant chemosensitivity in osteosarcoma.

## 1. Introduction

Osteosarcoma is one of the most common primary malignant bone tumors. Although neoadjuvant chemotherapy combined with surgical resection has significantly improved long-term survival in patients with localized disease, the heterogeneity of chemotherapy response among patients remains obvious [1,2]. At present, there is no global consensus on the definition and evaluation criteria of chemosensitivity [3,4]. This further limits the implementation of precision treatment strategies. Therefore, it is of great clinical significance to establish a noninvasive evaluation system that can predict neoadjuvant chemotherapy sensitivity at an early treatment stage or even before treatment. Such a system may optimize individualized treatment plans, avoid toxic effects caused by ineffective chemotherapy, and improve patient prognosis.

In recent years, the application of machine learning and radiomics in tumor treatment response prediction has developed rapidly [5,6,7,8]. Previous studies have shown that radiomics can predict the response of multiple solid tumors to chemotherapy, including ovarian cancer [9], esophageal cancer [10], and head and neck squamous cell carcinoma [11]. These studies generally believe that a large amount of hidden high-dimensional information in medical images can reflect potential biological characteristics such as necrosis degree and blood supply status, and these factors are closely related to chemotherapy sensitivity. Magnetic resonance imaging (MRI) is the most commonly used and most important imaging examination for osteosarcoma. It can noninvasively reflect the complexity of tumor internal structure. Methodologically, recent advances in MRI-based deep learning provide support for extracting complementary information beyond conventional radiomics. A comprehensive review and benchmarking study compared CNN and Transformer architectures for brain tumor classification in MRI, showing that hybrid CNN–Transformer models can integrate local texture features with global contextual information and improve classification robustness [12]. In addition, an automated IBNet9X–DenseNet8X framework for MRI-based heart disease diagnosis demonstrated the value of optimal feature fusion and densely connected networks for integrating complementary imaging features [13]. These methodological findings suggest that advanced deep learning and feature-fusion strategies can extract hidden high-dimensional information from MRI and provide a rationale for combining radiomics, clinical variables, and interpretability analysis in osteosarcoma. However, there are still few studies in the osteosarcoma field on MRI radiomics for predicting neoadjuvant chemotherapy sensitivity, especially studies that combine clinical variables and interpretability analysis.

In this study, we constructed an osteosarcoma neoadjuvant chemotherapy sensitivity prediction model based on MRI radiomics combined with clinical features, and further performed interpretability analysis at the image level and the feature level. Compared with relying only on traditional clinical indicators, the multimodal model constructed in this study can more comprehensively reflect the biological heterogeneity within the tumor. It provides a new idea for precision treatment of osteosarcoma.

## 2. Materials and Methods

### 2.1. Ethical Approval

This study was conducted in accordance with the Declaration of Helsinki and approved by the ethics committee 2025-922. In this retrospective study, the requirement for informed consent was waived. The study design followed the guidance of TRIPOD + AI.

### 2.2. Study Design

This study aimed to develop and validate a radiomics prediction model to predict the response of primary conventional osteosarcoma to chemotherapy. A total of 198 patients were included in this study from January 2016 to December 2025.

The inclusion criteria were as follows. First, patients with stage IIB primary high-grade conventional osteosarcoma confirmed by histopathological biopsy. Second, patients who completed a standard methotrexate, doxorubicin, and cisplatin (MAP) neoadjuvant chemotherapy regimen before definitive surgical resection. Specifically, we employed high-dose methotrexate (12 g/m^2^ IV over 4 h on day 1 with leucovorin rescue), doxorubicin (75 mg/m^2^ continuous IV infusion over 48 h starting day 8), and cisplatin (120 mg/m^2^ IV over 4 h on day 8 with aggressive hydration) in 28-day cycles. Patients received two preoperative cycles, with postoperative treatment adapted based on histologic response (continuing MAP for good responders or switching to MAPIE/HD-ifosfamide for poor responders) [14]. Third, patients with complete and high-quality baseline data, including prechemotherapy clinical demographic characteristics and serum indicators, and MRI data acquired before chemotherapy, including T1-weighted, T2-weighted, and contrast-enhanced sequences. The exclusion criteria were as follows. First, patients diagnosed with low-grade, parosteal, or periosteal osteosarcoma, or secondary osteosarcoma caused by Paget disease or previous radiotherapy. Second, patients with a history of other malignant tumors, or patients who had received nonstandard neoadjuvant therapy, local radiotherapy, or incomplete surgical intervention before enrollment. Third, cases with poor image quality, severe motion artifacts, metal artifacts, or missing sequences that prevented accurate delineation of the tumor region of interest (ROI). Fourth, cases with missing key clinical variables that could not be reasonably restored by imputation methods. Patients with ≥90% tumor necrosis were classified as chemotherapy-sensitive, and those with <90% necrosis as chemotherapy-insensitive. Patients were randomly divided into a training set and a test set with an 8:2 ratio. The split was conducted at the patient level. This approach prevented data leakage. All images, clinical variables, and repeated measurements from the same patient were assigned exclusively to one partition.

### 2.3. MRI Data Acquisition and Standardization

This study retrospectively retrieved and exported T2-weighted MRI data from the hospital picture archiving and communication system. All images were acquired using a 3.0 T magnetic resonance scanner, Siemens Trio Scanner system, Siemens Healthcare, Erlangen, Germany. The scan parameters were as follows. Slice thickness: 5 mm. Repetition time: 4000 ms. Echo time 93 ms. Echo train length: 1. Phase encoding percentage field of view: 80 percent. Matrix 320 by 230. Flip angle 120 degrees.

After the original digital Imaging and Communications in Medicine (DICOM) images were exported, two specialized physicians independently performed manual delineation of the tumor region of interest layer by layer on the axial plane. The delineation covered the level of the solid tumor component. In image quality control, cases with obvious motion artifacts, metal artifacts, or missing key sequences were excluded. To quantify the reproducibility of tumor delineation, inter-observer and intra-observer agreement were assessed in a randomly selected subset of *n* = 30 patients. Two musculoskeletal radiologists independently delineated the tumor ROI on the axial T2WI images while blinded to the pathological response and to all model outputs, and the first observer repeated the delineation after an interval of 4 weeks under identical display and window settings. Overlap between the two delineations was quantified with the Dice similarity coefficient (DSC), defined as DSC = 2 × |A ∩ B|/(|A| + |B|), where A and B denote the two ROI masks, and a DSC of ≥0.80 was prespecified as the threshold for acceptable agreement. Volume agreement was additionally evaluated with Bland–Altman analysis, in which the mean difference (bias) and the 95% limits of agreement (bias ± 1.96 SD) were calculated.

### 2.4. Image Preprocessing and Data Set Division

To evaluate the performance of multiple deep learning architectures in chemotherapy sensitivity prediction, this study first performed strict preprocessing and data set division on the original medical images. All input images were uniformly resized, and pixel-level grayscale values were normalized using standardized means and standard deviations to eliminate baseline distribution differences among samples. To improve model generalization and suppress overfitting caused by small samples, a highly randomized strong data augmentation strategy was introduced during model training. This included random horizontal and vertical flipping, affine transformation, color jitter, and Gaussian blur [15,16]. Specifically, the model input was a single axial T2WI slice corresponding to the maximum tumor cross-sectional area from the physician-delineated ROI, converted to a three-channel image by replicating grayscale values across RGB channels, resized to 224 × 224 pixels via bilinear interpolation, and z-score normalized using µ and σ derived solely from the training set and applied identically to validation and test sets; DICOM images were exported with the standard soft-tissue window preset from our PACS without additional manual windowing, consistent with current recommendations for reproducible radiomics preprocessing [17,18].

### 2.5. Deep Learning Model Architecture and Network Reconstruction

This study constructed a deep learning-based multimodel radiomics prediction framework for automatic binary classification of medical images. First, the original imaging data were organized into a unified data set format. Then, at the patient level, stratified random sampling was used to divide the data into a training set of 80 percent and a test set of 20 percent to ensure consistent class distribution and avoid data leakage.

All images were converted to red, green, and blue (RGB) format, uniformly resized to a fixed input size, and then standardized. Next, 12 classical convolutional neural network models were built, including DenseNet121, DenseNet169, DenseNet201 [19], ResNet18, ResNet34, ResNet50, ResNet101 [20], VGG16, VGG19 [21], EfficientNet B0, EfficientNet B4 [22], and MobileNetV2 [23]. All models loaded ImageNet pretrained weights for transfer learning. For different network structures, the classification head was redesigned. A dropout regularization layer and a nonlinear activation layer were added before the fully connected layer to reduce overfitting risk and enhance feature expression ability.

Model training used the AdamW optimizer and a cosine annealing learning rate scheduling strategy, and optimization was based on cross-entropy loss combined with label smoothing. For the ResNet series and DenseNet series models, a Mixup data mixing strategy was further introduced to improve model robustness. All models were implemented based on the PyTorch deep learning framework and trained and inferred in a graphics processing unit (GPU) environment. The input image size was uniformly set to 224 by 224 pixels. The batch size was 8. The number of epochs was 10. The optimizer was AdamW. The initial learning rate was 1 times 10 to the power of minus 4. The weight decay coefficient was 1 times 10 to the power of minus 2. The cosine annealing learning rate decay strategy was used. The number of classes in the classification task was 2. The loss function was cross-entropy loss with label smoothing set to 0.25.

All images were resized to 224 × 224 pixels and normalized. The dataset was divided into training and test sets at an 80:20 ratio using stratified random sampling with a fixed random seed of 42. The training batch size was 8, the test batch size was 1, and all models were trained for 10 epochs. Optimization was performed using AdamW with an initial learning rate of 1 × 10^−4^ and a weight decay of 1 × 10^−2^, together with a cosine annealing learning rate scheduler (T_max = 10). The loss function was cross-entropy with label smoothing of 0.25. Training-time data augmentation included random horizontal flipping, random vertical flipping with a probability of 0.5, random affine transformation (±15° rotation, translation of 0.1, and scaling of 0.9–1.1), ColorJitter (brightness and contrast = 0.2), and Gaussian blur (kernel size 3; σ = 0.1–2.0). At test time, only resizing and normalization were applied. Mixup augmentation (α = 0.4) was additionally applied to ResNet18/34/50/101 and DenseNet121/169/201. All models were initialized with ImageNet-pretrained weights and evaluated on the independent test set, with 95% confidence intervals estimated using 1000 bootstrap resamples.

To compare model risk stratification and robustness across datasets, sample Odds Ratios (ORs) and their 95% Wald confidence intervals (CIs) were calculated from confusion matrices utilizing a Haldane-Anscombe correction to eliminate zero-cell instability and Woolf’s method with exponential back-transformation for standard error estimation. The dataset was partitioned at the patient level into a training set (80%) and a held-out test set (20%) by stratified random sampling. Model selection and hyperparameter optimization across the 12 architectures were performed exclusively within the training set through patient-level five-fold cross-validation. In each fold, 10% of the patients in the four training folds were held out as an internal validation subset for early stopping and hyperparameter tuning, after which the model was retrained on the complete set of four training folds and evaluated on the fifth, held-out fold. No image from the held-out fold was used for training, early stopping or hyperparameter selection, and the 20% test set was not involved in any step of model development. Discrimination, accuracy, sensitivity and specificity were calculated for each fold and summarized as the mean and 95% confidence interval across the five folds, and the architecture with the highest mean cross-validated AUC was selected as the final imaging model.

### 2.6. Assessment of Predictor Redundancy and Multicollinearity

Redundancy among the predictors entering the combined model, including the radiomics score, was assessed with Spearman correlation analysis and variance inflation factors (VIF). VIF was computed as 1/(1 − R^2^) from a linear regression of each predictor on all remaining predictors, and a VIF below 5 was prespecified as the threshold for acceptable collinearity.

### 2.7. Model Interpretability Analysis

To overcome the black box limitation of deep learning models in clinical decision-making, this study introduced an improved gradient-weighted class activation mapping method, Gradient-weighted Class Activation Mapping++ (Grad-CAM++), to perform spatial interpretability analysis of the model decision process [24]. After model training and inference, we extracted the feature maps of the deep convolutional kernels and their corresponding gradient backpropagation information. High-resolution class activation heat maps were generated by calculating the distribution of feature space weights.

After pixel-level pseudo-color fusion and edge enhancement processing with the original medical images, the heat maps could intuitively and accurately highlight the anatomical or pathological local regions that the model highly focused on when predicting chemotherapy sensitivity. In addition, to quantify the consistency of spatial feature perception across different network architectures, we further calculated the structural similarity index between the Grad-CAM heat maps generated by each model. This provided an objective evaluation dimension for assessing the biological rationality of deep learning models in capturing low-level visual features.

### 2.8. Logistic Regression Model and Feature Attribution Analysis

The SHapley Additive exPlanations (SHAP) method provides local and global explanations by calculating the contribution of each feature to the prediction result, thus improving model transparency and interpretability [25,26,27,28]. In this study, we used SHAP for interpretability analysis of each patient feature. This method is based on Shapley value theory in cooperative game theory. It decomposes the predicted value by quantifying the marginal contribution of each feature to the model prediction result.

### 2.9. Diagnostic Performance Evaluation

This study constructed a multidimensional statistical indicator system to evaluate the diagnostic performance and clinical application potential of the prediction models. The main quantitative evaluation indicators included the area under the receiver operating characteristic curve, area under the curve (AUC), precision–recall curve area, average precision (AP), accuracy, sensitivity, specificity, F1 score, and odds ratio. To objectively quantify the statistical fluctuation range of model performance, the bootstrap resampling method was used to calculate nonparametric confidence intervals for each evaluation indicator.

In addition, calibration curves were drawn to strictly evaluate the goodness of fit between model output probability and the true observed risk. Radar charts, violin plots of predicted probability distribution, and bubble charts of model complexity and performance were also used to visualize the overall diagnostic ability of the models from multiple angles.

For statistical significance testing, the DeLong test was introduced to perform paired verification of the AUC distributions of different networks in the test set, and the Z statistic and corresponding *p*-value reflecting model performance differences were calculated. This study first evaluated the imaging prediction model. After selecting the best model, it was fused with clinical indicators. Then the multimodal model and the single modality model were evaluated for performance heterogeneity.

### 2.10. Statistical Analysis

All statistical analyses and clinical data visualizations were performed using R software (version 4.3.1; R Foundation for Statistical Computing, Vienna, Austria) within the RStudio environment (version 2023.09.1+494; Posit Software, PBC, Boston, MA, USA). Deep learning model construction, image preprocessing, data augmentation, transfer learning, Grad-CAM++ visualization, SHAP interpretability analysis, and machine learning workflows were implemented in Python (version 3.10.13; Python Software Foundation, Wilmington, DE, USA) using PyTorch (version 2.1.0), Scikit-learn (version 1.3.2), NumPy (version 1.26.2), Pandas (version 2.1.4), Matplotlib (version 3.8.2), Seaborn (version 0.13.0), and SHAP (version 0.44.1) libraries. Continuous variables with a normal distribution are presented as mean ± standard deviation (SD) and were compared using Student’s *t*-test. Continuous variables with a non-normal distribution are presented as median with interquartile range (IQR) and were compared using the Mann–Whitney U test or Kruskal–Wallis H test. Categorical variables are presented as counts and percentages and were compared using the chi-square test or Fisher exact test. Pearson and Spearman correlation analyses were used to evaluate relationships between variables. Model discrimination was assessed using the area under the receiver operating AUC, AP, accuracy, sensitivity, specificity, and F1 score. Calibration was evaluated using calibration curves, while clinical utility was assessed using decision curve analysis (DCA). DeLong tests, net reclassification improvement (NRI), and integrated discrimination improvement (IDI) were used to compare predictive performance between models [29]. Statistical significance was defined as a two-sided *p*-value < 0.05. All methods are described in detail in the corresponding Results sections and figure legends.

## 3. Results

### 3.1. Dataset Characteristics

**Figure 1:**
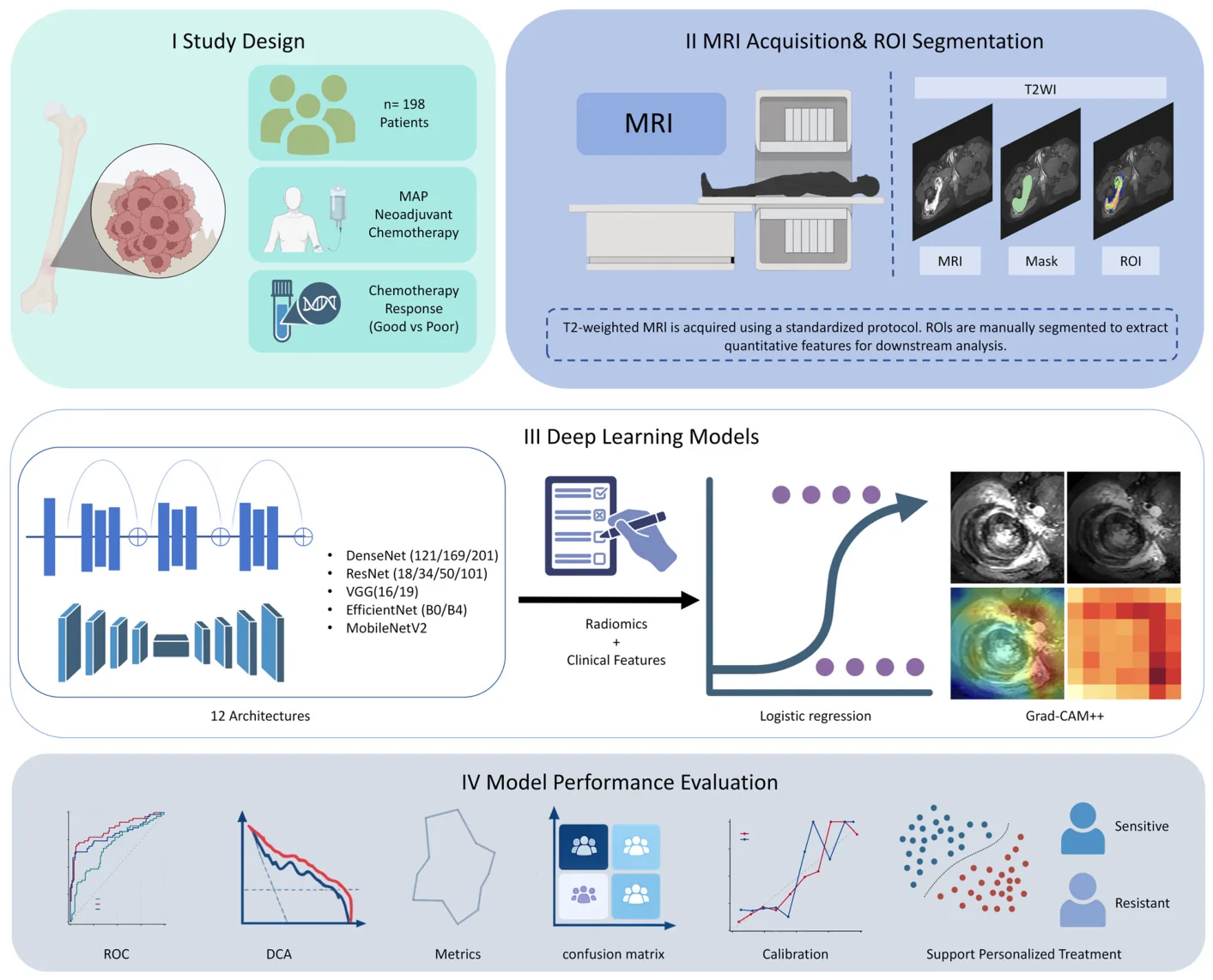
Patient screening flow chart.

The study flow is shown in Figure 1. A total of 198 patients with stage IIB conventional osteosarcoma were finally included, including 125 patients in the chemotherapy-insensitive group and 73 patients in the chemotherapy-sensitive group (Supplementary Figure S1A). Most demographic characteristics and peripheral serum indicators were balanced between the two groups, and no significant differences were observed (Table 1).

Patients who responded favorably to neoadjuvant chemotherapy had significantly higher body mass index (BMI) (23.604 ± 6.684 vs. 20.850 ± 6.698, *p* = 0.003) and smaller maximum tumor diameters (5.126 ± 2.927 cm vs. 6.565 ± 3.024 cm, *p* < 0.001) compared with non-responders. These results indicate that better nutritional status and reduced tumor burden are potentially associated with enhanced chemosensitivity.

### 3.2. Deep Learning Model Screening and Performance Comparison

Before model training, inter- and intra-observer agreement in region-of-interest delineation was assessed using the DSC. DSC values generally exceeded the prespecified threshold of 0.80 and ranged from approximately 0.80 to 0.95. Intra-observer agreement showed a slightly higher median DSC than inter-observer agreement. These findings supported the reproducibility of the imaging annotations used for subsequent model development (Supplementary Figure S2).

**Figure 2:**
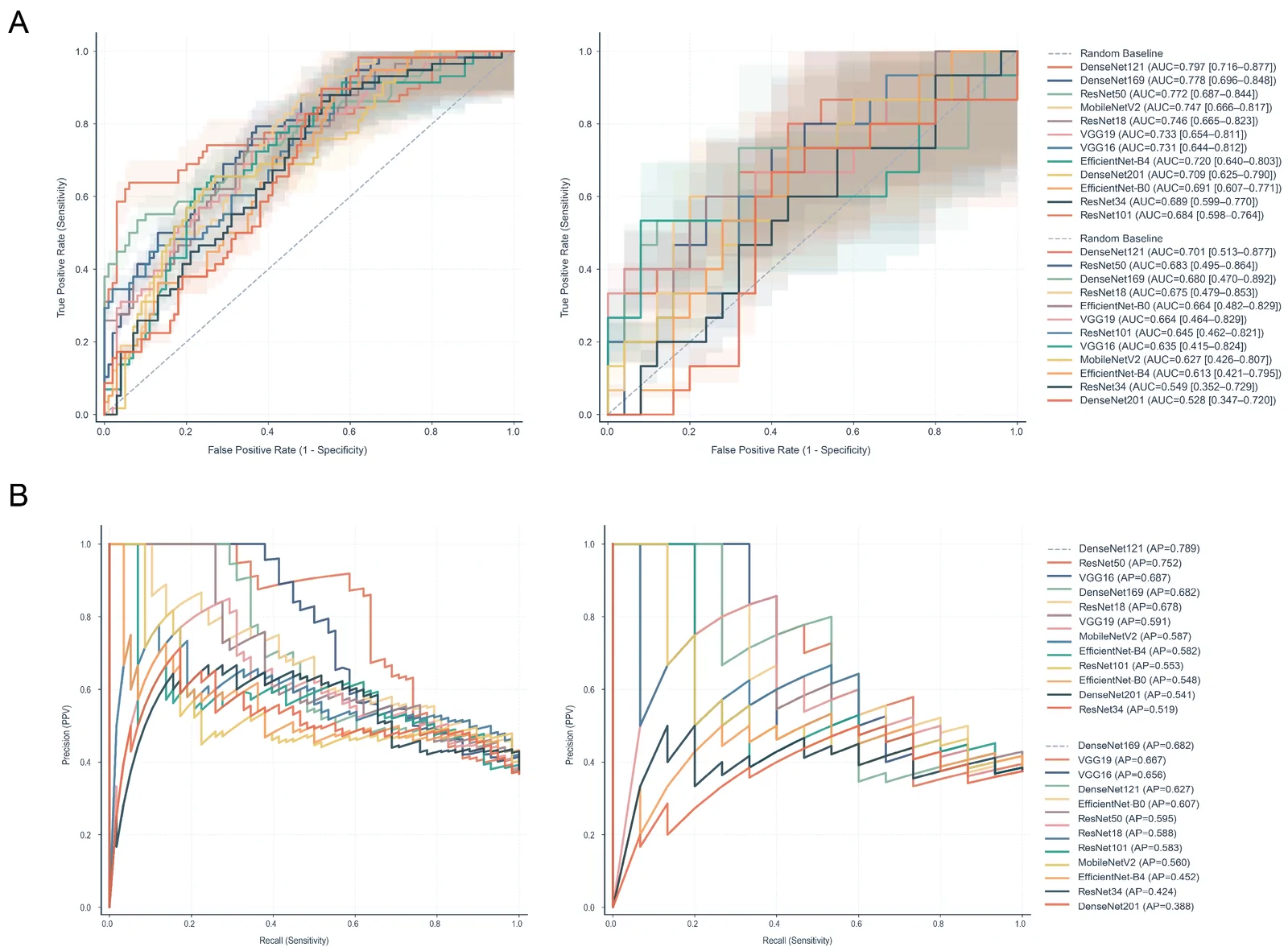
Discrimination kinetics and precision–recall trajectories. (**A**) ROC curves comparing the predictive performance of the 12 deep learning architectures in the training cohort ((**left**) panel) and testing cohort ((**right**) panel). (**B**) PR curves comparing the positive prediction performance of the 12 deep learning architectures in the training cohort ((**left**) panel) and testing cohort ((**right**) panel).

Following these standardized preprocessing and reliability validations, images entered the model training process. To systematically evaluate the predictive ability of different deep learning architectures for chemosensitivity, this study constructed and compared 12 mainstream deep learning models based on the T2WI sequence. Receiver Operating Characteristic (ROC) curve and precision–recall curve analyses showed that DenseNet121 performed best among all candidate models (Figure 2).

In the training set, DenseNet121 achieved an accuracy of 0.789, sensitivity of 0.690, specificity of 0.800, and F1 score of 0.678. The area under the receiver operating characteristic curve (AUC) was 0.797 (95% CI, 0.716–0.877), and average precision (AP) was 0.789. In the test set, DenseNet121 achieved a sensitivity of 0.867 for identifying chemotherapy-sensitive patients and an F1 score of 0.619. The AUC was 0.701 (95% CI, 0.513–0.877), and AP was 0.627. Despite higher sensitivity, the lower test-set AUC indicated reduced discrimination compared with the training set (Figure 2). Five-fold cross-validation complemented the training–test evaluation and informed model selection by assessing performance across multiple data partitions. DenseNet121 achieved the highest mean AUC among the 12 candidate models (0.775), with a mean specificity of 0.890. These findings supported selecting DenseNet121 for subsequent model development, although the wide test-set confidence interval limits conclusions about its generalizability (Supplementary Figure S3).

The confusion matrix analysis further verified the above results (Supplementary Figures S4–S6). DenseNet121 maintained a high correct classification proportion in both the training set and the test set, while most other models showed obvious overfitting, with high performance in the training set and significantly decreased performance in the test set.

**Figure 3:**
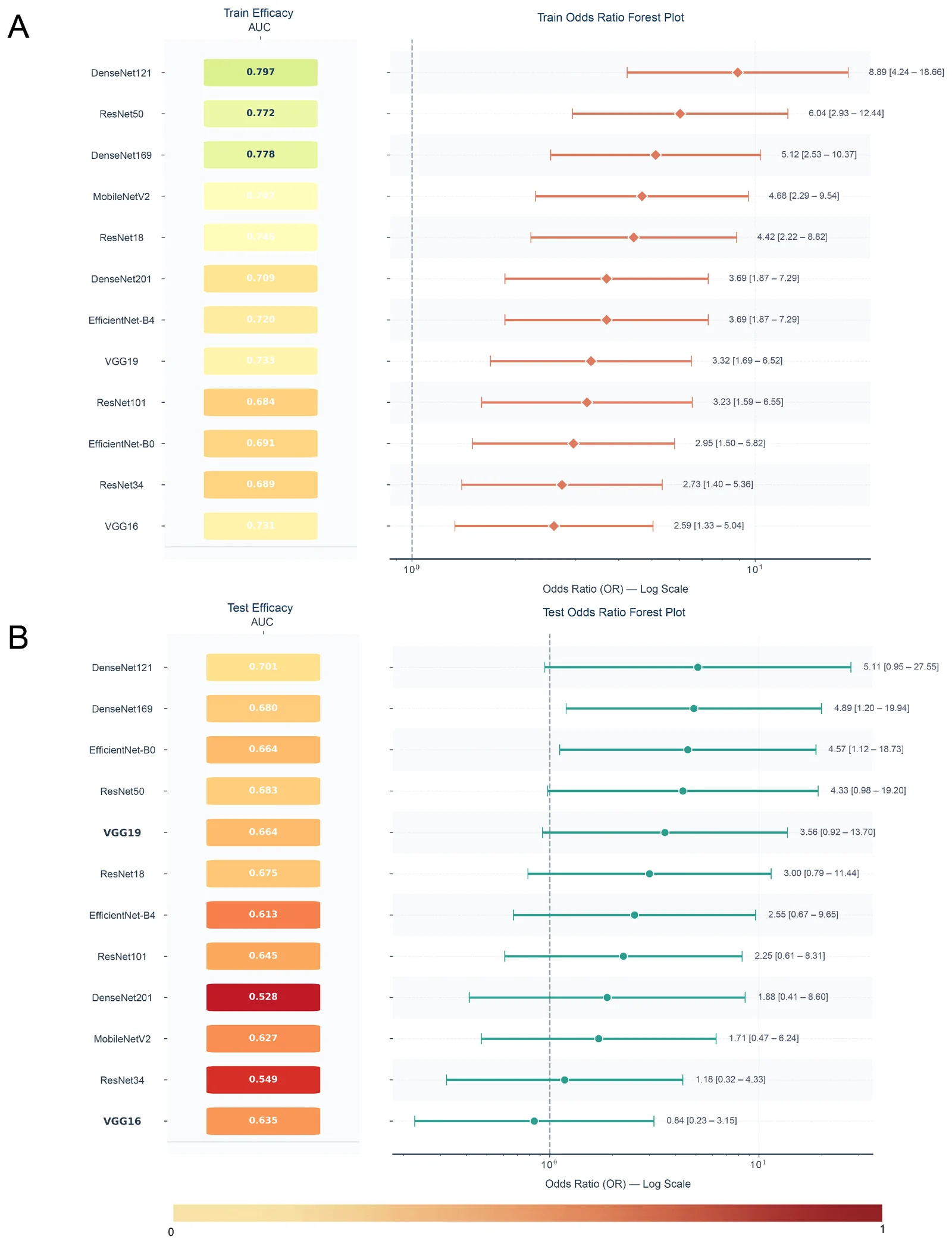
Efficacy performance validation and risk odds ratio profiles. (**A**) Forest plot showing the AUC, OR, and corresponding 95% CI of the 12 deep learning models in the independent training cohort. (**B**) Forest plot showing the testing AUC performance and OR distribution of the 12 deep learning models in the training cohort. The color gradient represents the model performance metric, ranging from 0 to 1.

To further evaluate risk stratification, we computed ORs between predicted risk groups. The training set yielded an OR of 8.89 (95% CI: 4.24–18.66, *p* < 0.001), confirming strong discriminative power. The test set showed an OR of 5.11 (95% CI: 0.95–27.55, *p* = 0.056), indicating a consistent trend though not reaching statistical significance, likely due to the smaller validation cohort. Overall, DenseNet121 effectively differentiated patients with high versus low probability of chemotherapy sensitivity, supporting its clinical utility (Figure 3).

### 3.3. Interpretability Analysis of the Radiomics Prediction Model

**Figure 4:**
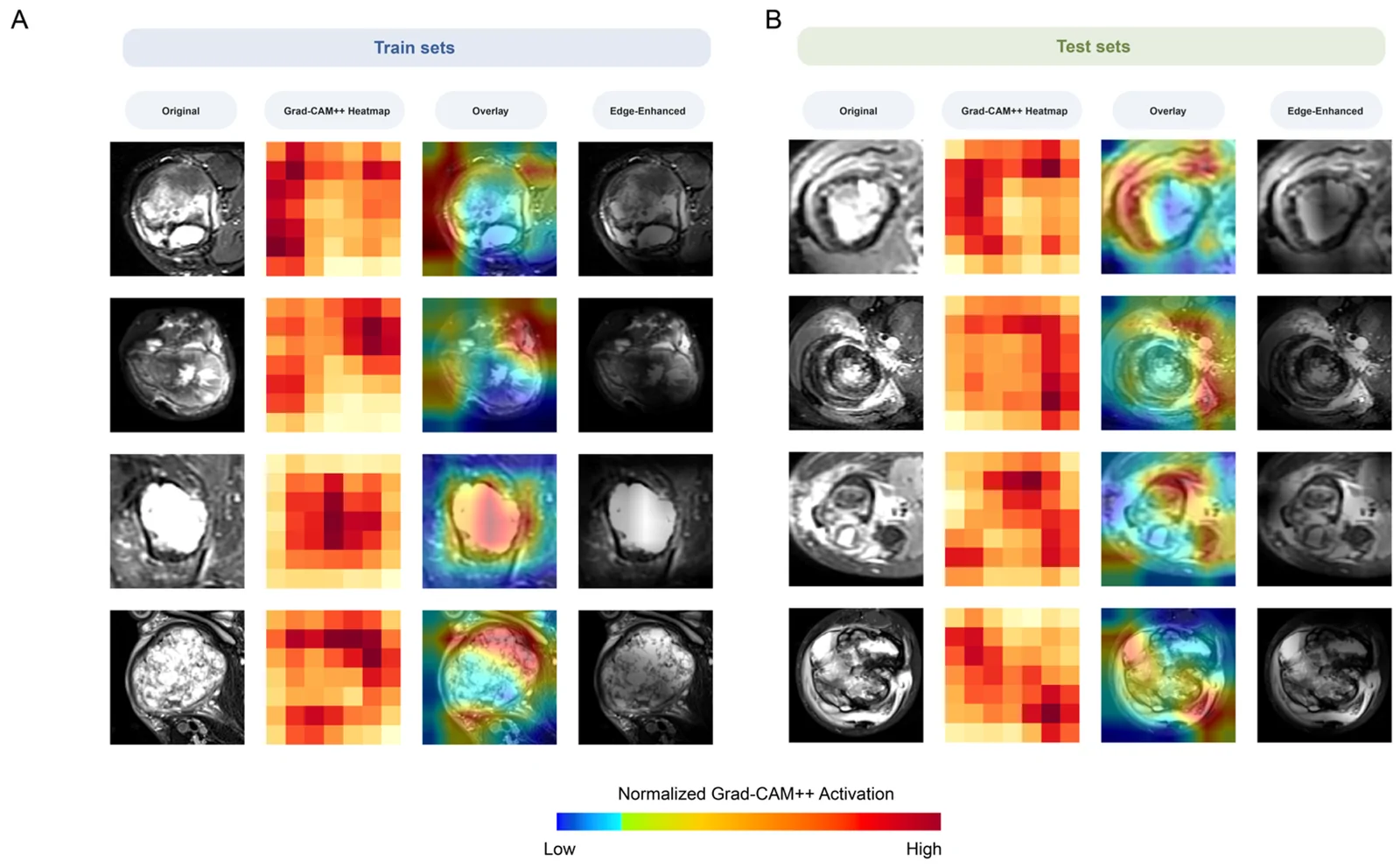
Spatial visual explainability mapping via Grad-CAM++ of DenseNet121. (**A**) Representative Grad-CAM++ visual explanations of DenseNet121 in the training cohort. (**B**) Representative Grad-CAM++ visual explanations of DenseNet121 in the testing cohort. Each panel contains four complementary views: the original T2-weighted MRI image, the Grad-CAM++ activation heatmap, the pseudocolor overlay image, and the edge-enhanced visualization image.

To explore the decision basis of the deep learning model, we further performed interpretability analysis. The heat map results showed that DenseNet121 mainly focused on the solid infiltrative area inside the osteosarcoma lesion and the extraosseous soft tissue invasion area (Figure 4). These areas usually represent the most biologically active parts of the tumor and are also important imaging regions for clinical evaluation of tumor invasiveness.

It is worth noting that the high-weight regions focused on by different deep learning models were highly consistent (Supplementary Figure S7). Most models focused attention on the center of the tumor, the necrosis active boundary region, and the tumor margin region. This suggests that the key features captured by deep learning models may come from the biological information reflected by tumor internal heterogeneity and its invasive front.

### 3.4. Clinical Feature Screening

**Figure 5:**
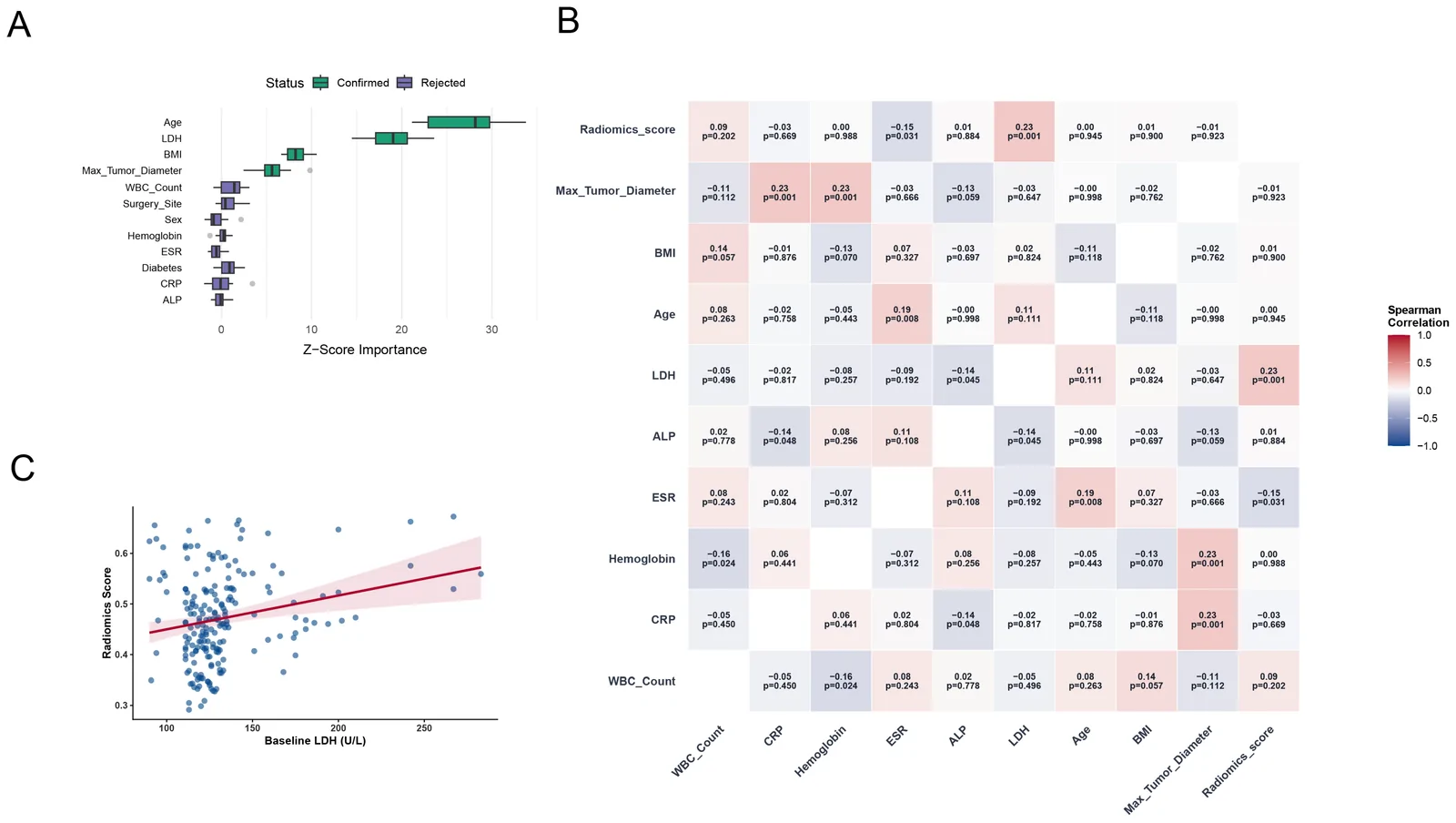
Clinical feature screening and multimodal correlation matrix analysis in osteosarcoma. (**A**) Boruta feature selection analysis showing the importance distribution of clinical and serological variables. Green indicates confirmed features, whereas blue indicates rejected features. (**B**) Heatmap showing the correlation matrix between radiomics score and clinical variables. Correlation coefficients and corresponding *p*-values are displayed in each cell. (**C**) Scatter plot showing the association between baseline serum LDH and radiomics score, with fitted regression line. The gray dots represent outliers.

To further improve prediction performance and build a multimodal model, we included clinical variables and radiomics features in the joint analysis. Boruta feature selection showed that age, LDH, BMI, and maximum tumor diameter were the most predictive clinical variables (Figure 5A). These variables were retained for subsequent joint model construction. VIF analysis (Supplementary Figure S8) demonstrated that all retained variables exhibited low collinearity (VIF range: 1.09–1.52), confirming the robustness of the joint model construction. To avoid data leakage and ensure unbiased evaluation, the Boruta feature selection was performed exclusively on the training set, and the selected features were then applied to the validation and test sets.

Then, we further analyzed the correlation between the radiomics score and the clinical indicators. The correlation matrix and scatter fitting analysis showed that the radiomics score was significantly positively correlated with baseline LDH level (Spearman *r* = 0.23, *p* = 0.001) (Figure 5B,C). LDH is an important metabolic indicator of tumor glycolytic activity. Its elevation usually suggests an enhanced Warburg effect in tumor cells. The positive correlation between the radiomics score and LDH suggests that the high-dimensional imaging features extracted by deep learning may reflect osteosarcoma metabolic activity and the microenvironment heterogeneity induced by it. This finding supports the potential link between deep learning features and tumor biological behavior at the imaging level [30].

### 3.5. Construction of the Combined Model and Evaluation of Predictive Performance

**Figure 6:**
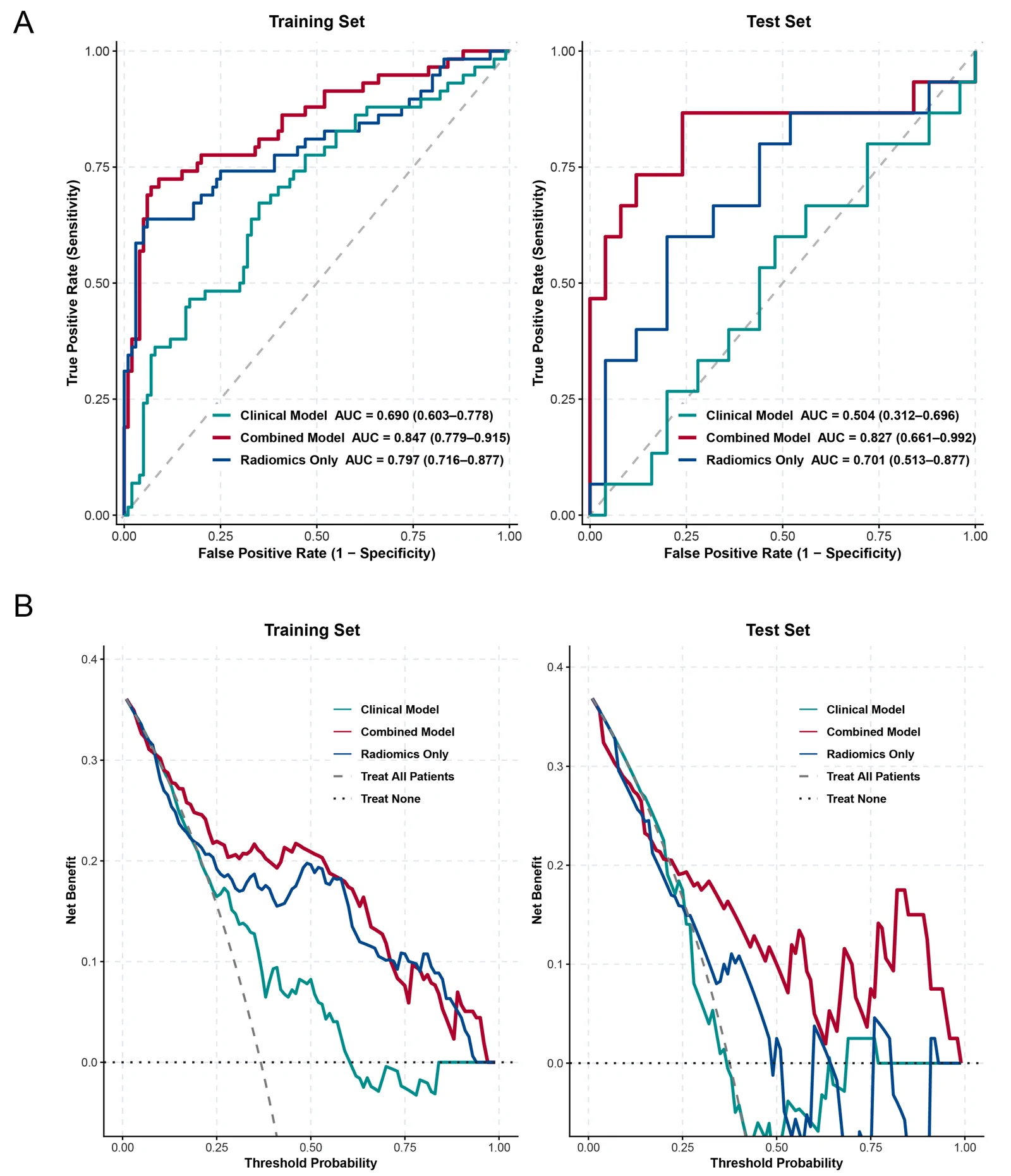
Multimodal predictive fusion and clinical DCA. (**A**) ROC curves comparing the discrimination performance of the multimodal combined model and the radiomics-only model in the training and testing cohorts. (**B**) DCA evaluating the clinical net benefit of each model in the training cohort ((**left**) panel) and testing cohort ((**right**) panel).

Based on the selected clinical variables and radiomics score, we constructed a logistic regression combined prediction model. ROC analysis demonstrated that the combined model exhibited superior discriminatory power compared to the single radiomics model and clinical model alone (Figure 6A). In the training set, the combined model achieved an AUC of 0.847 (95% CI: 0.779–0.915), markedly outperforming the radiomics-only model (AUC: 0.797, 95% CI: 0.716–0.877) and the clinical model (AUC: 0.690, 95% CI: 0.603–0.778). In the test set, the combined model maintained robust predictive performance with an AUC of 0.827 (95% CI: 0.661–0.992), which was notably higher than the radiomics-only model (AUC: 0.701, 95% CI: 0.513–0.877) and the clinical model (AUC: 0.504, 95% CI: 0.312–0.696). Decision curve analysis (Figure 6B) further revealed that the combined model yielded a higher net benefit across a wide range of threshold probabilities in both cohorts. These results indicate that clinical information provides a crucial supplement to imaging features, thereby enhancing the model’s ability to identify chemotherapy sensitivity.

### 3.6. Reclassification Benefit Analysis

To evaluate the incremental value of the combined model relative to the single radiomics and clinical models, we further performed model improvement index analysis. The DeLong test demonstrated statistically significant improvements in AUC for the combined model in both cohorts. Specifically, compared with the radiomics-only model, the DeLong *p*-values were 0.0458 (training set) and 0.0107 (test set); compared with the clinical model, the DeLong *p*-values were 0.001 (training set) and 0.0409 (test set), suggesting the combined model achieved a real and reliable performance gain. NRI and IDI analyses further corroborated this finding. In the training set, the combined model yielded an NRI of 0.6559 (95% CI: 0.3492–0.9626, *p* < 0.001) and an IDI of 0.084 (95% CI: 0.0358–0.1321, *p* = 0.0006) over the radiomics model, and an NRI of 0.9738 (95% CI: 0.6887–1.2589, *p* < 0.001) with an IDI of 0.2923 (95% CI: 0.2113–0.3733, *p* < 0.001) over the clinical model. In the test set, the corresponding NRI and IDI for the combined versus radiomics model were 1.28 (95% CI: 0.7835–1.7765, *p* < 0.001) and 0.1765 (95% CI: 0.1025–0.2505, *p* < 0.001), respectively, while versus the clinical model they were 0.7733 (95% CI: 0.2520–1.2947, *p* = 0.0036) and 0.326 (95% CI: 0.1774–0.4746, *p* < 0.001). Additionally, compared with the clinical model, the radiomics model significantly improved risk reclassification and discrimination as measured by NRI and IDI in the training set (NRI, 0.7193; 95% CI, 0.4182–1.0204; *p* < 0.001; IDI, 0.2084; 95% CI, 0.1097–0.3070; *p* < 0.001). However, the difference in AUC did not reach statistical significance (DeLong *p* = 0.0985). In the test set, neither the reclassification and discrimination improvements nor the AUC difference were statistically significant (NRI, 0.1867; 95% CI, −0.3981–0.7714; *p* = 0.5315; IDI, 0.1495; 95% CI, −0.0068–0.3059; *p* = 0.0609; DeLong *p* = 0.1892) (Table 2).

### 3.7. SHAP Interpretability Analysis of the Combined Model

**Figure 7:**
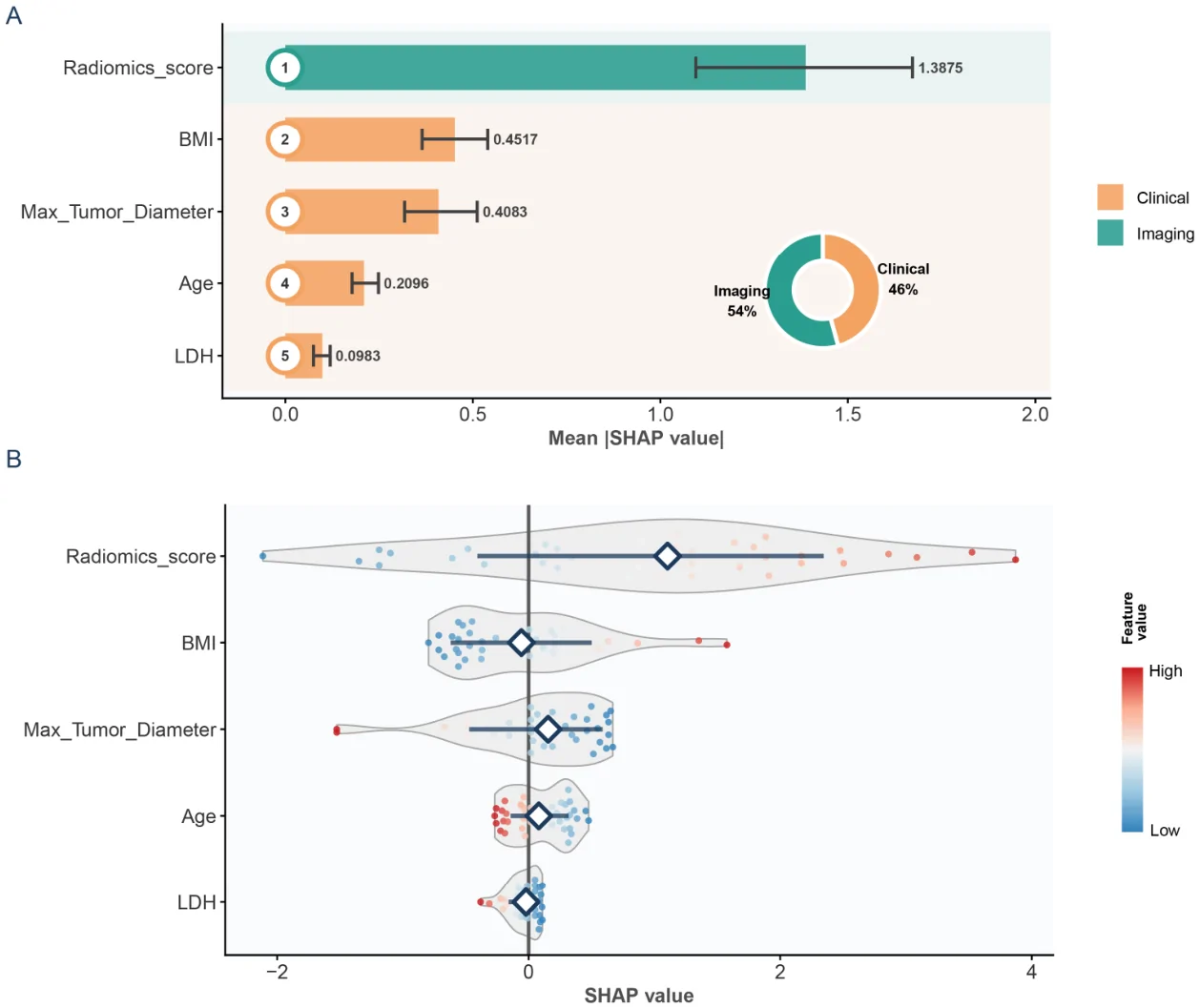
Feature attribution metrics via SHAP interpretability. (**A**) Global feature importance plot showing the mean absolute SHAP value of each predictor in the combined model. The embedded pie chart illustrates the relative contributions of clinical variables and radiomics score. (**B**) SHAP summary beeswarm plot showing the directional effects of individual feature values on model prediction probability.

To clarify the contribution of each variable to the decision process of the combined model, we used the SHAP algorithm for global and local interpretation analysis. Global contribution analysis showed that the imaging modality contributed 54 percent of the overall decision weight, while clinical variables contributed 46 percent (Figure 7A). This indicates that radiomics information is still the core source driving model prediction, but clinical information also plays an important supporting role.

Feature importance ranking showed that the radiomics score was the most important predictive factor in the combined model, with a mean absolute SHAP value of 1.3875 (Figure 7B). It was followed by a BMI of 0.4517, a maximum tumor diameter of 0.4083, an age of 0.2096, and LDH of 0.0983. Local interpretability analysis further showed that a high radiomics score was significantly associated with a favorable chemotherapy response. At the same time, a smaller maximum tumor diameter also had a positive effect on chemosensitive outcome. Interestingly, when a patient had both a higher radiomics score and a lower initial tumor burden, the probability of obtaining a good histological response increased significantly.

The above results showed that the combined model not only improved overall discrimination ability, but also significantly improved patient risk stratification and individualized classification accuracy. Compared with the single radiomics model, the combined model could more accurately identify potential chemotherapy-sensitive patients and chemotherapy-insensitive patients, thereby providing a more reliable basis for treatment decision-making.

## 4. Discussion

This study constructed and validated a noninvasive prediction system for neoadjuvant chemotherapy sensitivity in osteosarcoma based on deep learning multimodal feature fusion. By performing parallel benchmarking across 12 classical convolutional neural network backbones, we established DenseNet121 reconstructed from the T2WI sequence as the optimal single-modality imaging radiomics core. We then refined age, baseline lactate dehydrogenase, BMI, and maximum tumor diameter as the core clinical risk variables using the Boruta algorithm. The results showed that the AUC of the test set increased from 0.701 for the single imaging model to 0.827.

When our model was compared with nontraditional methods, the prediction model combined with radiomics data showed significantly improved performance. This was consistent with reports of radiomics prediction models in other solid tumors [10,31,32,33]. A recent meta-analysis involving 1639 patients showed that the pooled AUC of radiomics models in the training set and validation set reached 0.93 and 0.87, respectively [34], fully demonstrating the overall predictive potential of this field. Models using the T2WI sequence generally showed performance between 0.71 and 0.76 [35,36]. Models based on DWI radiomics had an AUC of about 0.77 [37]. Different from these studies, our study used deep learning for prediction, which can further mine information in the images and achieve better predictive performance. In addition, in this study, the radiomics score accounted for 54 percent of the core decision weight. After deep fusion with clinical variables such as age, BMI, and maximum tumor diameter, the test AUC of the combined model increased significantly from 0.701 to 0.827. This gain from multimodal combination is highly consistent with the findings of Luo et al., combined model AUC 0.828 [38], and Zheng et al., nomogram AUC 0.815 [39], and is also consistent with the overall trend in the meta-analysis that combined clinical and imaging models are better than single radiomics models [34].

A major drawback of radiology models is the lack of interpretability, which limits clinical application to some extent. Doctors need to clearly understand how the model reaches its results and identify key radiological features in the decision process. This is a challenge. Previous studies have mainly used Grad CAM and its derivatives for interpretability analysis of radiomics images, including hepatocellular carcinoma [40,41], glioblastoma [42,43], and rectal cancer [44]. In this study, Grad CAM++ visually confirmed that the DenseNet121 network consistently focused on the medullary solid infiltrative area and the extraosseous soft tissue invasion area in both the training set and the test set. This conclusion was consistent with the core regions of previous glioma studies, suggesting that medical deep learning networks may have an intrinsic mathematical property of spontaneously capturing the most invasive core pathological microenvironment with the highest cell density, often accompanied by microvascular abnormality and heterogeneous hypoxia, when mining malignant tumors of different anatomical sites and histological origins [44].

Although single-sequence T2WI is widely available, robust to acquisition differences, and captures tumor extent, intratumoral heterogeneity, and peritumoral changes, it lacks the perfusion, permeability, and cellularity information provided by dynamic contrast-enhanced T1WI and diffusion-weighted imaging, which have demonstrated value in osteosarcoma response prediction [37,45]. Deep feature extraction can partially compensate for this limitation by encoding subtle textural, spatial, and peritumoral heterogeneity that conventional visual assessment and classical radiomics may overlook [37]. Nevertheless, such compensation is not equivalent to direct functional assessment, and integrating DCE and DWI into deep-learning frameworks remains an important future direction [37,45].

Several limitations should be acknowledged when interpreting the present findings. First, this was a single-center retrospective study of 198 patients with stage IIB conventional osteosarcoma, and all evaluation was internal. Model development, architecture selection and threshold definition were performed within the same cohort, and the 20% test set, although not used for model selection, contained only approximately 40 patients. The resulting confidence intervals were therefore wide. Case mix, chemotherapy adherence, scanner type and imaging parameters are also likely to differ elsewhere, so the present performance may not transfer to other institutions, and external multicenter validation remains necessary before clinical use. Second, only the T2WI sequence was used for imaging prediction. T2 hyperintensity reflects viable tumor together with peritumoral edema, inflammation and joint effusion, and it provides no direct measure of perfusion, membrane permeability or cellularity. The absence of dynamic contrast-enhanced and diffusion-weighted data may therefore reduce the specificity of the radiomics score and limit its biological interpretability. Third, response was defined by a binary histological threshold, which is subject to inter-observer variability. Patients whose necrosis fraction lies close to the cutoff may be misclassified, and the two classes were imbalanced. Fourth, Grad-CAM++ was applied qualitatively. It showed which tumor regions drove model decisions, but peak activation was not quantified against pathologically verified margins, and attention to irrelevant background could not be excluded. Quantifying localization accuracy by Dice overlap with expert annotations, and testing stability by perturbation analysis, are therefore required. Finally, the radiomics score was not linked to genomic or transcriptomic data, so its biological basis remains unclear. Clinical utility was demonstrated only by decision curve analysis, not by a prospective impact study. Future work should therefore combine multi-sequence MRI, pathology and multi-omics data within a multicenter prospective design, in order to clarify the mechanisms underlying the radiomics score and to establish its clinical value. Collectively, these limitations highlight the exploratory nature of the present study, and the findings should be interpreted with caution before any clinical translation is considered.

## 5. Conclusions

In conclusion, this study established and validated an explainable, multimodal fusion framework integrating T2WI deep learning radiomics and clinical features to predict neoadjuvant chemosensitivity in stage IIB conventional osteosarcoma. By fusing the optimal DenseNet121 architecture with core clinical variables, the combined model significantly enhanced predictive performance and risk stratification over single-modality methods. Furthermore, Grad-CAM++ and SHAP interpretability successfully resolved the deep learning “black box” limitation, demonstrating high clinico-pathological transparency. This noninvasive framework offers a robust auxiliary decision-support tool to guide pre-treatment individualized precision therapy and improve patient prognoses.

## Figures and Tables

**Table 1:** Patient baseline demographic and clinical characteristics.

Characteristic	N	Overall	No	Yes	*p*-Value
		N = 198	N = 125	N = 73	
WBC Count	198	7.642 ± 3.880	7.661 ± 4.289	7.608 ± 3.085	0.679
CRP	198	7.844 ± 22.908	8.988 ± 24.435	5.885 ± 20.036	0.177
Hemoglobin	198	111.242 ± 20.599	110.672 ± 19.947	112.219 ± 21.775	0.739
ESR	198	33.011 ± 25.063	33.337 ± 25.237	32.452 ± 24.927	0.858
ALP	198	85.697 ± 31.097	86.800 ± 33.946	83.808 ± 25.603	0.638
LDH	198	132.177 ± 29.765	133.544 ± 29.944	129.836 ± 29.514	0.688
Age	198	21.924 ± 8.911	23.056 ± 9.464	19.986 ± 7.549	0.036
BMI	198	21.865 ± 6.808	20.850 ± 6.698	23.604 ± 6.684	0.003
Sex	198				0.759
Female		114 (58%)	73 (58%)	41 (56%)	
Male		84 (42%)	52 (42%)	32 (44%)	
Max Tumor Diameter	198	6.034 ± 3.061	6.565 ± 3.024	5.126 ± 2.927	<0.001
Surgery Site	198				0.432
Clavicle		1 (0.5%)	1 (0.8%)	0 (0%)	
Femur		93 (47%)	54 (43%)	39 (53%)	
Fibula		3 (1.5%)	1 (0.8%)	2 (2.7%)	
Humerus		33 (17%)	20 (16%)	13 (18%)	
Ilium		1 (0.5%)	1 (0.8%)	0 (0%)	
Metacarpal		1 (0.5%)	1 (0.8%)	0 (0%)	
Patella		1 (0.5%)	1 (0.8%)	0 (0%)	
Pelvis		39 (20%)	28 (22%)	11 (15%)	
Scapula		6 (3.0%)	2 (1.6%)	4 (5.5%)	
Talus		1 (0.5%)	1 (0.8%)	0 (0%)	
Tibia		18 (9.1%)	14 (11%)	4 (5.5%)	
Ulna		1 (0.5%)	1 (0.8%)	0 (0%)	
Diabetes	198	48 (24%)	34 (27%)	14 (19%)	0.204

**Table 2:** Model improvement metrics.

Comparison	Dataset	NRI_Value	NRI_95CI	NRI_P	IDI_Value	IDI_95CI	IDI_P	DeLong_P
Combined vs. Clinical	Train	0.9738	[0.6887, 1.2589]	0	0.2923	[0.2113, 0.3733]	0	0.001
Combined vs. Clinical	Test	0.7733	[0.2520, 1.2947]	0.0036	0.326	[0.1774, 0.4746]	0	0.0409
Combined vs. Radiomics	Train	0.6559	[0.3492, 0.9626]	0	0.084	[0.0358, 0.1321]	0.0006	0.0458
Combined vs. Radiomics	Test	1.28	[0.7835, 1.7765]	0	0.1765	[0.1025, 0.2505]	0	0.0107
Radiomics vs. Clinical	Train	0.7193	[0.4182, 1.0204]	0	0.2084	[0.1097, 0.3070]	0	0.0985
Radiomics vs. Clinical	Test	0.1867	[−0.3981, 0.7714]	0.5315	0.1495	[−0.0068, 0.3059]	0.0609	0.1892

## Data Availability

The code supporting this study is available at https://github.com/Jianhua-Mu/Deep-Learning-Radiomics-for-Osteosarcoma-Chemotherapy-Response-Using-T2WI-and-Clinical-Data (accessed on 15 March 2026). Further inquiries can be directed to the corresponding authors.

## Supplementary Materials

The following supporting information can be downloaded at: https://www.mdpi.com/article/10.3390/diagnostics16203411/s1, Supplementary Figure S1. Screening flowchart of osteosarcoma patients and MRI tumor ROI segmentation. Supplementary Figure S2. Assessment of region of interest (ROI) segmentation consistency using the Dice Similarity Coefficient. Supplementary Figure S3. Performance comparison of 12 deep learning architectures under five-fold cross-validation. Supplementary Figure S4. Confusion matrices of twelve deep learning model architectures in training and testing cohorts. Supplementary Figure S5. Radar plot performance evaluation of twelve deep learning frameworks in training and testing cohorts. Supplementary Figure S6. Bar chart comparison of multidimensional diagnostic metrics for twelve deep learning models in training and testing cohorts. Supplementary Figure S7. Visual interpretability analysis of deep learning models using Grad-CAM++ activation mapping. Supplementary Figure S8. VIF analysis for assessing multicollinearity among clinical variables. TRIPODAI checklist.pdf [46,47].
