# Supplementary material for: Fusion of Deep Learning Radiomics and Clinical Features for Individualized Prediction of Neoadjuvant Chemotherapy Response in Osteosarcoma
Source: Diagnostics (Basel). 2026 Oct 20;16(20):3411. doi: 10.3390/diagnostics16203411 (PMC13703618; doi:10.3390/diagnostics16203411)
Supplement: Supplementary file 1 [file diagnostics-16-03411-s001.zip › Supplementary materials.pdf]

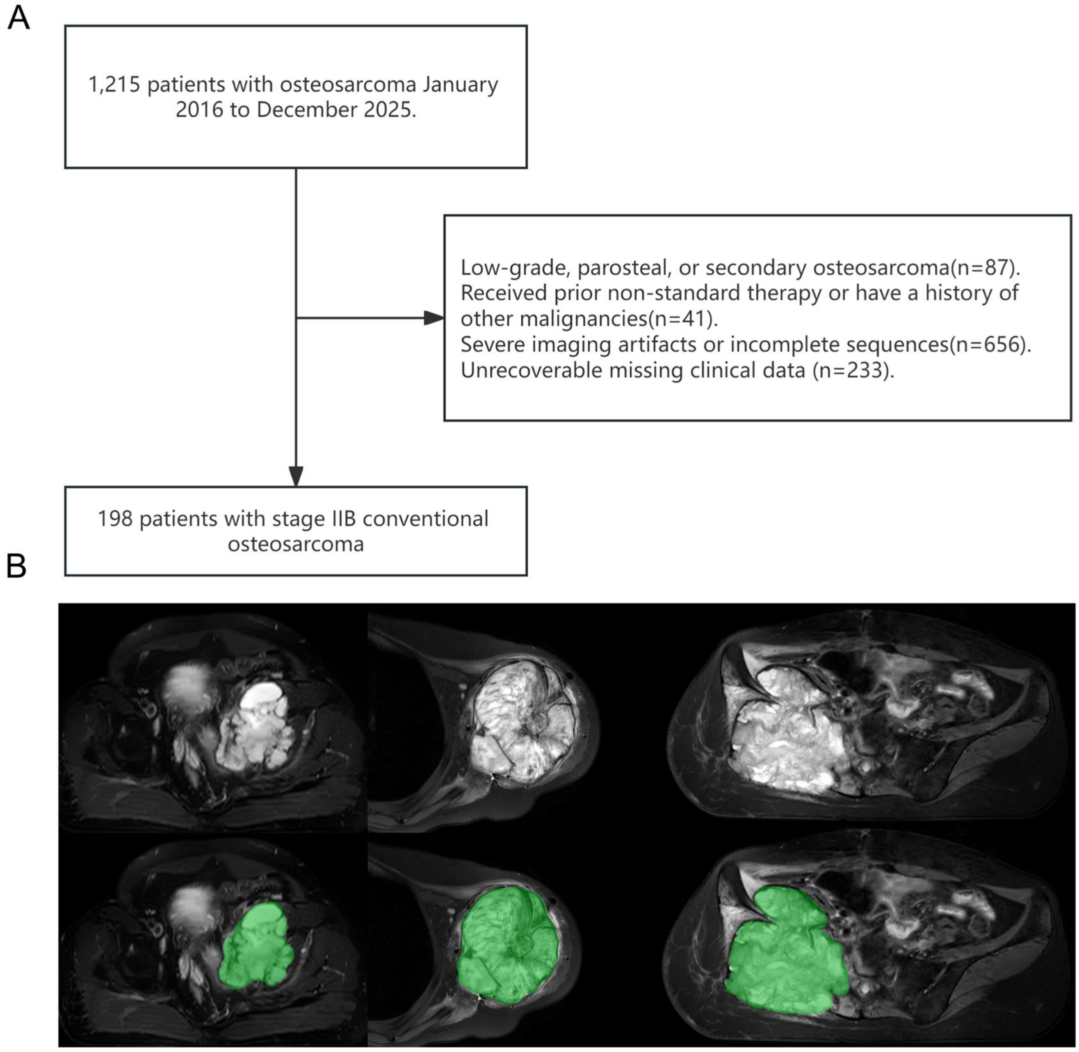

Figure S1. Screening flowchart of osteosarcoma patients and MRI tumor ROI segmentation. (A) Representative axial T2-weighted MRI images and corresponding manual delineation of tumor ROIs. (B) Study population screening flow chart.

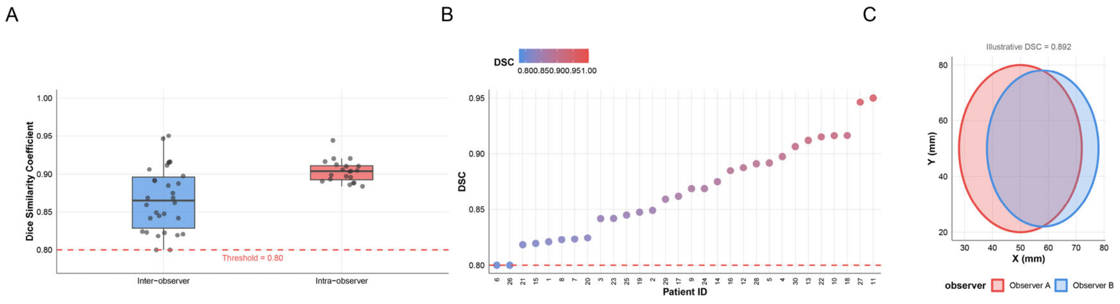

Figure S2. Assessment of region of interest (ROI) segmentation consistency using the DSC. (A) Box plot showing the distribution of inter-observer and intra-observer DSC, with a consistency threshold of 0.80 (dashed red line); intra-observer agreement is slightly higher than inter-observer agreement. (B) Scatter plot of inter-observer DSC per patient sorted by Patient ID, colored by DSC magnitude, indicating consistent overlap across most subjects. (C) Schematic diagram illustrating ROI overlap between Observer A and Observer B, with an illustrative DSC of 0.892.

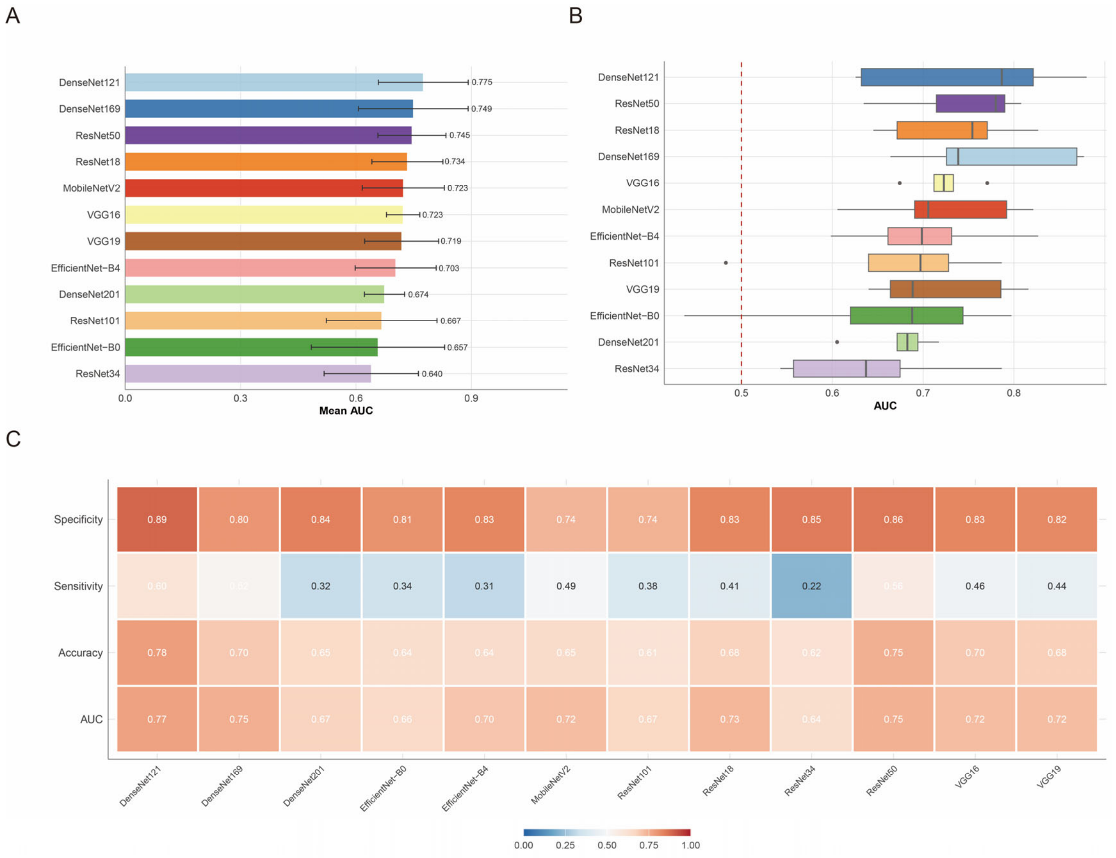

Figure S3. Performance comparison of 12 deep learning architectures under five-fold cross-validation. (A) Horizontal bar plot of mean AUC values ranked in descending order, with DenseNet121 achieving the highest mean AUC of 0.775; error bars indicate variability across folds. (B) Box plots illustrating the distribution of AUC values across the five folds for each model, with the red dashed line denoting the chance level (AUC = 0.5). (C) Heatmap summarizing the mean specificity, sensitivity, accuracy, and AUC across the five folds.

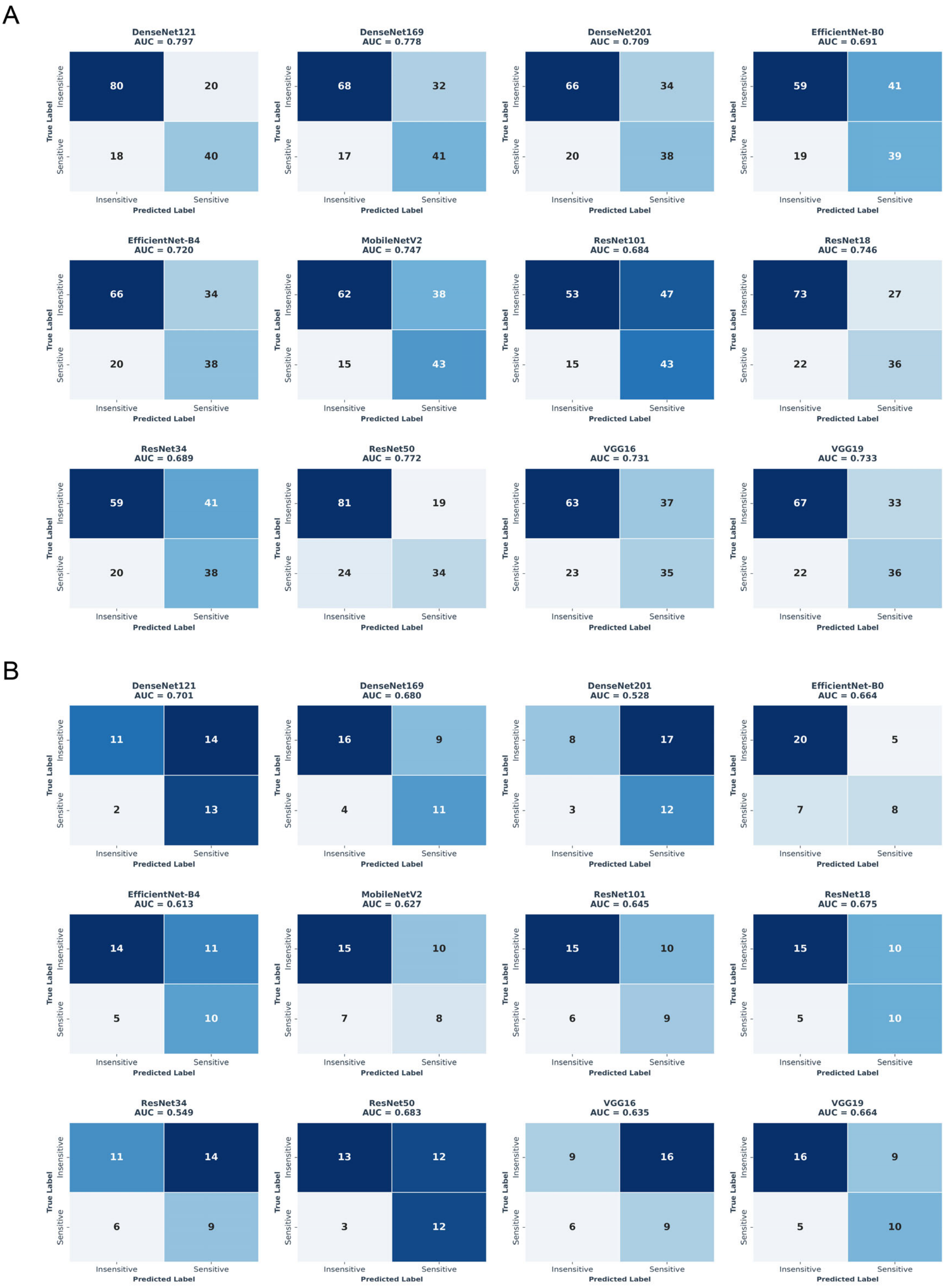

Figure S4. Confusion matrix arrays across twelve deep learning model architectures. (A) Confusion matrices of the 12 deep learning models in the training cohort. (B) Confusion matrices of the 12 deep learning models in the testing cohort.

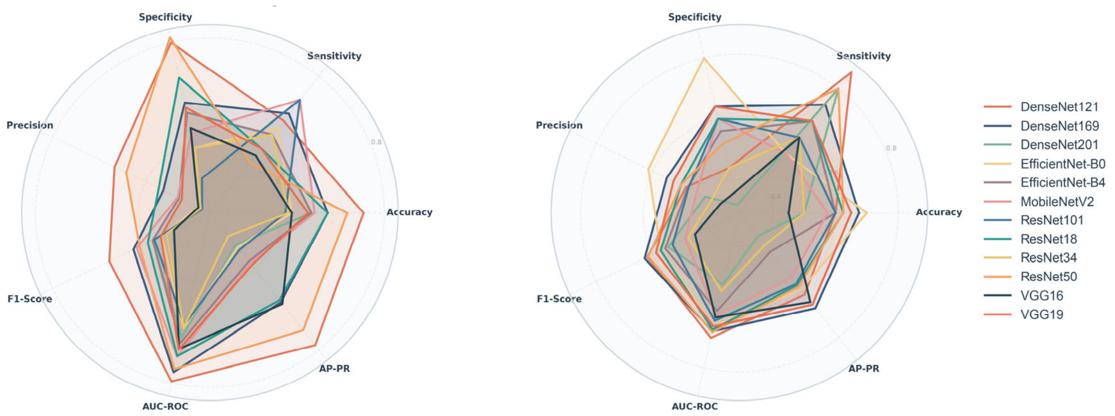

Figure S5. Radar topography performance envelopes for deep learning frameworks. (A) Radar plots summarizing the predictive performance of the 12 deep learning models in the training cohort. (B) Radar plots summarizing the predictive performance of the 12 deep learning models in the testing cohort.

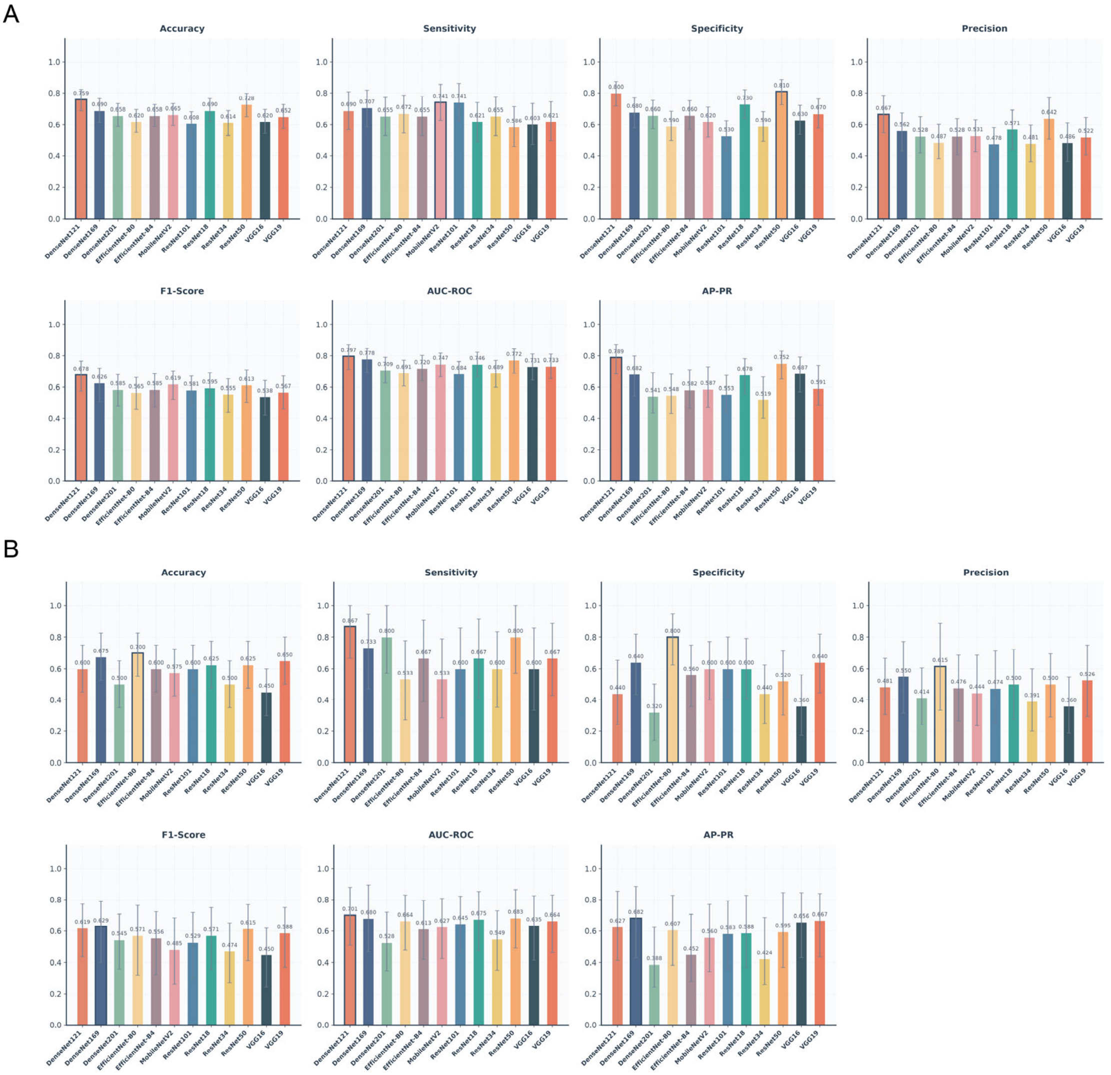

Figure S6. Multidimensional diagnostic matrix and classification efficacy bars. (A) Grouped bar plots summarizing accuracy, sensitivity, specificity, precision, F1-score, AUC-ROC, and AP-PR of the 12 deep learning models in the training cohort. (B) Comparative bar plots showing the corresponding performance metrics in the testing cohort.

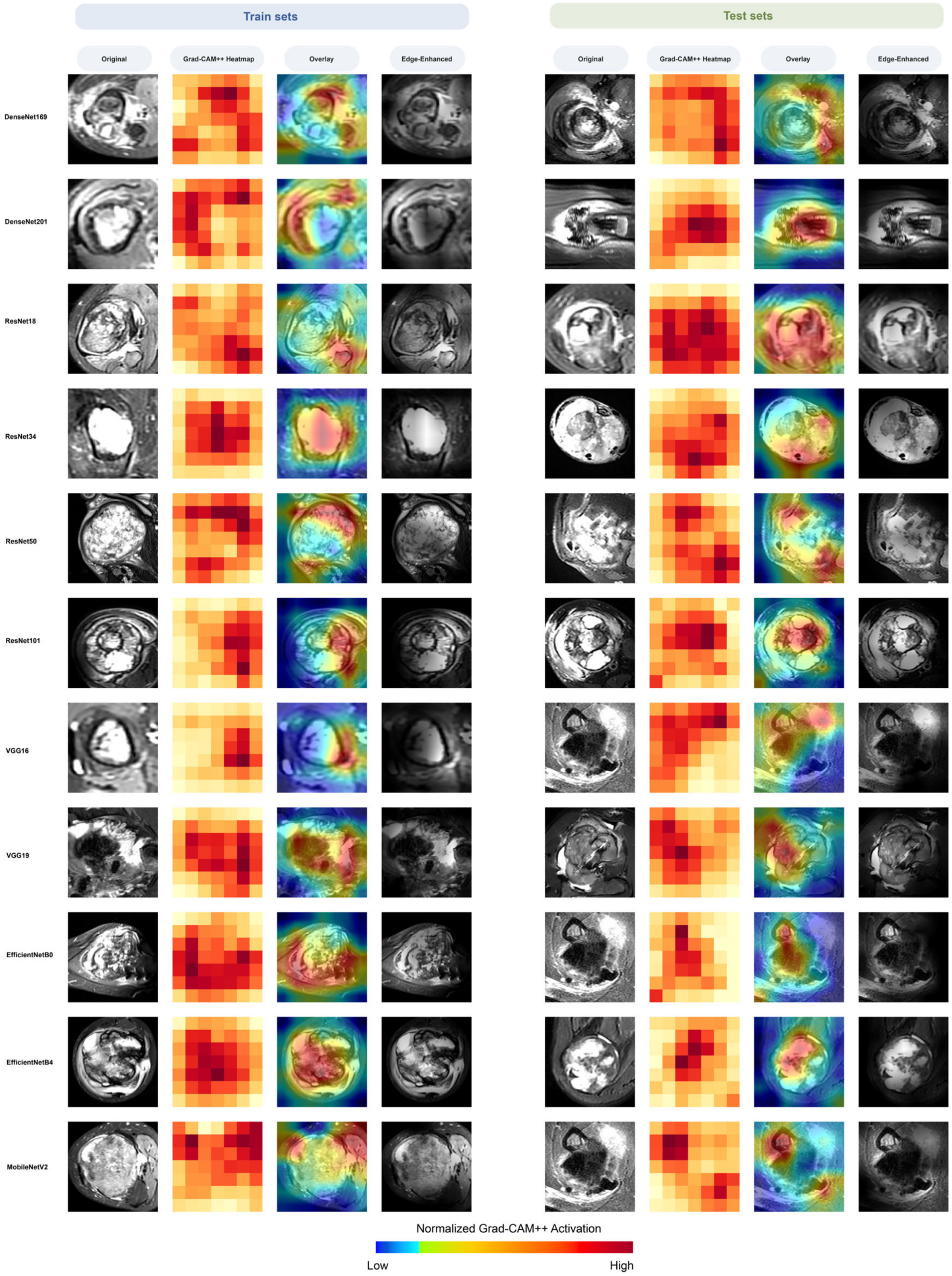

Figure S7. Spatial visual explainability mapping via Grad-CAM++. (A) Grad-CAM++ activation maps of 11 deep learning models in the training cohort. (B) Grad-CAM++ activation maps of the same models in the testing cohort. Each model unit contains four complementary views: the original T2-weighted MRI image, Grad-CAM++ activation heatmap, pseudocolor overlay image, and edge-enhanced visualization image.

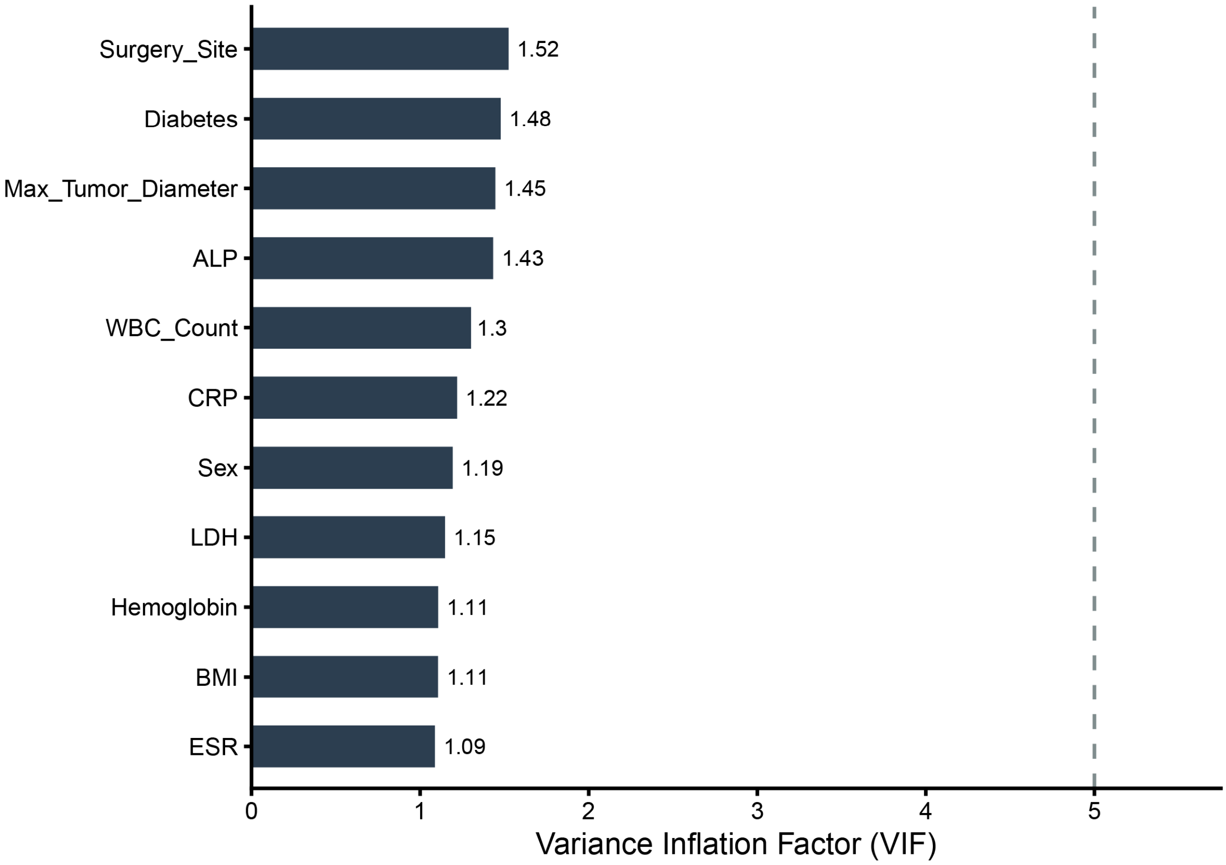

Figure S8. VIF analysis for assessing multicollinearity among clinical variables.
